# Microbial community structure and functional potential of tropical lithic habitats in northern Thailand

**DOI:** 10.3389/fmicb.2026.1794540

**Published:** 2026-05-12

**Authors:** Sahassawat Chailungka, Kodchaporn Khamkak, Arita Intanon, Senoch Seanpong, Jaturong Kumla, Nakarin Suwannarach, Terd Disayathanoowat, Nuttapol Noirungsee

**Affiliations:** 1 Master of Science Program in Applied Microbiology (International Program), Faculty of Science, Chiang Mai University, Chiang Mai, Thailand; 2 Department of Biology, Faculty of Science, Chiang Mai University, Chiang Mai, Thailand; 3 Bioinformatics and Systems Biology Program, School of Bioresources and Technology and School of Information Technology, King Mongkut’s University of Technology Thonburi, Bangkok, Thailand; 4 Institute of Plant Sciences and Microbiology, Department of Biology, Universität Hamburg, Hamburg, Germany; 5 Center of Excellence in Microbial Diversity and Sustainable Utilization, Chiang Mai University, Chiang Mai, Thailand; 6 Office of Research Administration, Chiang Mai University, Chiang Mai, Thailand

**Keywords:** biogeochemical process, extreme environment, lithic habitat, lithic microbial community, tropical ecosystem

## Abstract

Rock (lithic) habitats are macroscopically inhospitable, characterized by oligotrophic conditions and exposure to environmental extremes across diverse climate regions. However, at the microscale, lithic substrates provide sheltered niches, such as pores, cracks, and fissures, that buffer external stressors and enable microbial persistence. While lithic microbial communities have been extensively studied in extreme climates, those in tropical regions remain underexplored. This study investigated epilithic communities on sandstone, as well as both epilithic and endolithic communities associated with limestone in northern Thailand. Bulk soil samples in the immediate vicinity of the lithic substrates were examined to compare microbial community differences between lithic and soil environments. Actinobacteria, Cyanobacteria, and Proteobacteria dominated in bacterial communities across lithic habitats and substrates, while Acidobacteria were more prevalent in soil communities. Lithic fungal communities were predominantly composed of lichen-forming fungi, including Dothideomycetes, Eurotiomycetes, and Lecanoromycetes. Differential abundance analysis revealed that photosynthetic bacteria, radio-resistant bacteria, and rock-inhabiting fungi (RIF) were predominant in the lithic communities. Metabolic functions related to oxygenic photoautotrophy were significantly enriched, corresponding with the high abundance of photosynthetic cyanobacteria. These cyanobacteria contribute to carbon fixation through photosynthesis and to nitrogen fixation, thereby enhancing nutrient availability for microbes that depend on organic carbon. Furthermore, functional predictions suggest that MND1, IS-44, mle1-7, Ellin6067, and SC-I-84 potentially contribute to ammonia oxidation and nitrate generation, while *Nitrospira* supports nitrification and *Rhodoplanes* facilitates denitrification. The presence of phosphate-solubilizing and siderophore-producing bacteria suggests active nutrient mobilization, supporting microbial community resilience and contributing to nutrient cycling and ecosystem functioning within lithic habitats. These findings suggest that lithic ecosystems in tropical regions represent ecologically unique niches that harbor microorganisms capable of facilitating nutrient cycling, thereby supporting their colonization. Moreover, this study highlights the untapped biodiversity of microbes in the tropical regions of northern Thailand.

## 1 Introduction

In exposed environments such as deserts, alpine regions, or other areas with extreme climates, microorganisms face multiple stresses, including intense solar radiation, frequent desiccation and rehydration, temperature fluctuations, and nutrient limitation (Gorbushina, 2007). Lithic habitats provide sheltered microenvironments that mitigate these conditions and support microbial persistence (Choe et al., 2018; Ertekin et al., 2021; Fernández-Martínez et al., 2021). Structural features such as pores and fissures further enhance habitability by reducing radiation exposure, retaining moisture, and buffering temperature changes (Choe et al., 2021; Ertekin et al., 2021; Meslier et al., 2018). Microbial survival in lithic environments relies on adaptations such as tolerance to resource limitation, resistance to environmental stress, and flexible growth strategies (Choe et al., 2018).

Microbial communities in lithic habitats, particularly hypolithic and endolithic systems, have been relatively well studied and are often structured around primary producers that sustain ecosystem functioning. Photoautotrophic and diazotrophic microorganisms, including algae and Cyanobacteria, play key roles by supplying organic carbon through photosynthesis and contributing to nitrogen inputs, thereby supporting associated heterotrophic communities (Ertekin et al., 2021; Fernández-Martínez et al., 2021; Meslier and DiRuggiero, 2019; Zhao et al., 2023). In addition, the production of extracellular polymeric substances (EPS) enhances moisture retention, promotes adhesion to mineral surfaces, and stabilizes microhabitats during hydration cycles, facilitating the persistence and activity of microbial consortia (Meslier and DiRuggiero, 2019; Zhao et al., 2023).

In contrast, epilithic microbial communities have received comparatively less focused attention and are frequently grouped within broader lithic or endolithic contexts. In lithic ecosystems, many microorganisms exhibit ecological flexibility, occupying both surface (epilithic) and subsurface (endolithic) niches depending on environmental conditions, resulting in partial taxonomic and functional overlap. However, recent evidence suggests that even millimeter-scale environmental gradients can lead to distinct community composition and diversity, reflecting niche-specific adaptation (Stoppiello et al., 2024). These differences are likely driven by variations in light exposure, moisture availability, and mineral interactions at the rock surface. Therefore, explicitly investigating epilithic habitats is essential to resolve fine-scale ecological differentiation and to better understand how microorganisms adapt to surface-associated stresses.

Lithic microbial communities have been investigated across a wide range of rock types, including sandstone, limestone, gypsum, dolomite, ignimbrite, granite, halite, and basalt, in diverse extreme environments such as hyper-arid deserts, polar regions, and high-altitude ecosystems (Choe et al., 2018; Coleine et al., 2021b; Meslier et al., 2018). These studies demonstrate that substrate properties play a critical role in shaping microbial community structure and function. Physicochemical characteristics such as mineral composition, porosity, permeability, surface morphology, and light transmissivity influence water retention, nutrient availability, and microclimatic stability, thereby affecting microbial colonization and community assembly (Choe et al., 2018). Microbial colonization of lithic substrates has also been reported in culturally significant stone monuments, where it contributes to biodeterioration and material alteration. For example, high-throughput sequencing analyses of ancient stone sculptures in China revealed diverse microbial communities whose composition was strongly influenced by environmental factors such as light, humidity, and air pollutants, with phototrophs and nitrifying bacteria playing key roles in surface degradation processes (Li et al., 2016).

Despite these advances, lithic microbial communities in tropical regions remain comparatively underexplored. Tropical environments, often recognized as biodiversity hotspots, may harbor distinct lithic microbial assemblages shaped by unique climatic conditions. In northern Thailand, recent studies have reported novel rock-inhabiting fungi associated with sandstone substrates (Thitla et al., 2023), highlighting the potential for discovering previously uncharacterized microbial diversity in this region.

In this study, sandstone and limestone were selected as representative lithic substrates due to their contrasting physicochemical properties and ecological relevance. Sandstone is typically characterized by higher porosity and permeability, which can enhance water retention and provide protected microhabitats, whereas limestone is dominated by carbonate minerals and exhibits different surface chemistry and dissolution dynamics that influence microbial colonization. By comparing these substrates, this study aims to assess how rock type influences microbial diversity and community composition in tropical lithic environments.

This study investigated the microbial communities associated with two distinct lithic substrates (sandstone and limestone) in northern Thailand. Functional activity within the lithic habitats was assessed to explore their ecological roles. The objectives of this study were twofold: (1) to characterize the bacterial and fungal communities and their diversity within lithic environments in northern Thailand, with emphasis on their ecological significance; and (2) to infer the potential functional roles of microbial communities in key biogeochemical processes within these lithic habitats under the prevailing climatic conditions.

## 2 Materials and methods

### 2.1 Sample collection and sample preparation

**Figure 1:**
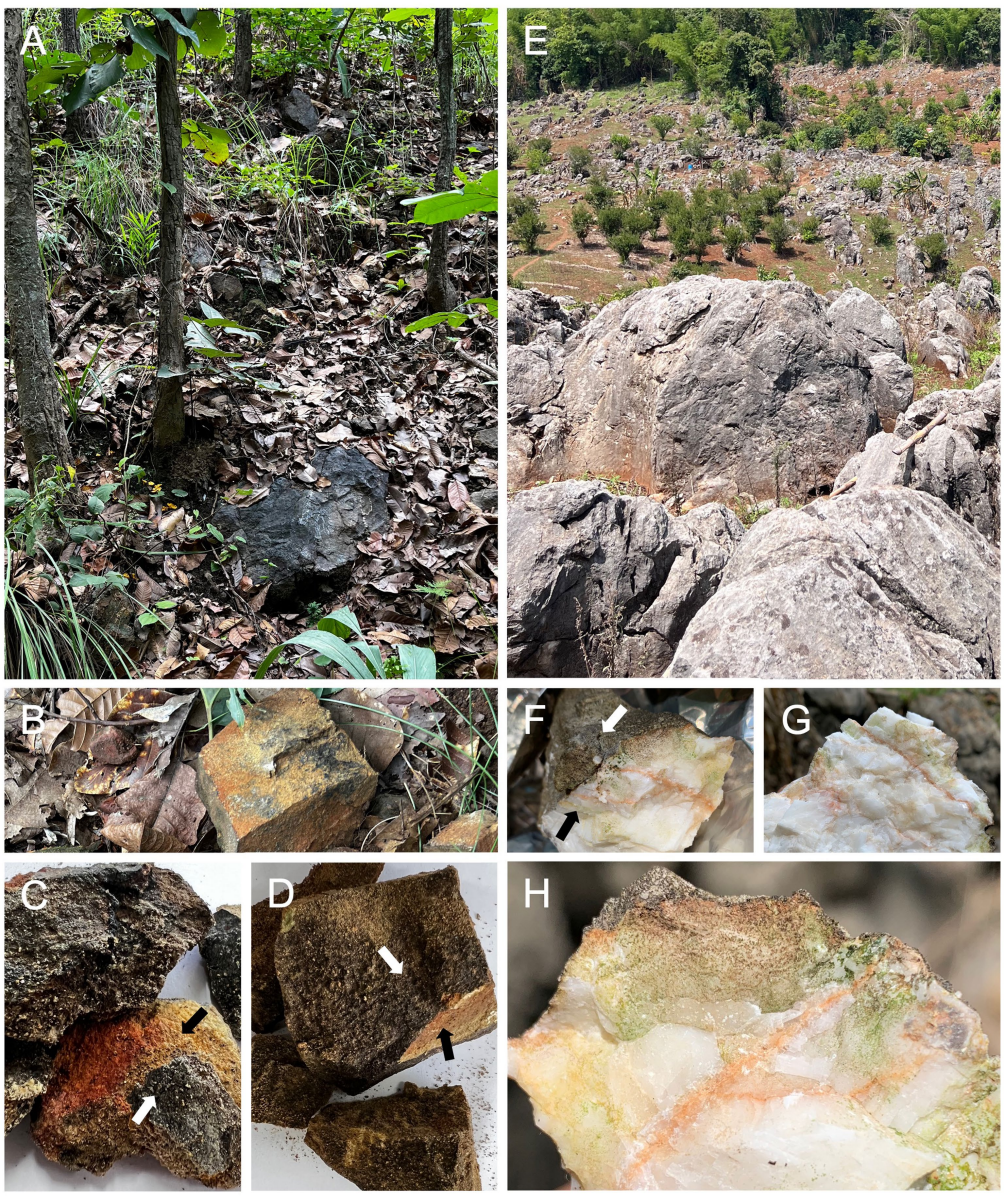
Sampling sites and representative lithic substrates. **(A–D)** Sandstone: **(A)** Sandstone sampling site; **(B)** fractured sandstone at the sampling site; **(C, D)** fractured sandstone samples. **(E–H)** Limestone: **(E)** Limestone sampling site; **(F–H)** fractured limestone samples showing visible green biofilm/plaque within the rock. White arrows indicate the rock surface, and black arrows indicate the interior of the rock.

Rock specimens were collected from northern Thailand. Specifically, 10 samples from a sandstone site were obtained from Chiang Mai University’s “Hariphunchai” Center in Lamphun Province (coordinates: 18.51206°N, 99.11372°E) in August 2022. The environmental conditions during collection included an average temperature of 26.0 °C and a relative humidity of 93.5%. The sandstone site was characterized by vegetation cover including grasses, leaf litter, and tree canopy (Figure 1). Additionally, 10 samples from a limestone site were collected in June 2023 from Chai Prakan District, Chiang Mai Province (coordinates: 19.70670°N, 99.03056°E). Environmental conditions at the limestone site included an average temperature of 27.5 °C and a relative humidity of 58.0%. The limestone site was exposed to direct sunlight (Figure 1). Specimens were selectively collected based on visual inspection, ensuring no observable mosses or lichens were present, and were preserved by wrapping them in sterile aluminum foil. Epilithic microbial communities were collected on-site by swabbing a 2 × 2 cm^2^ area from the surface of each rock and immediately preserved in DNA/RNA Shield Reagent (Zymo Research, Irvine, CA). To ensure sample integrity, all specimens were immediately transported to the laboratory. Both rock specimens and surface samples intended for microbial community analysis were preserved at −20 °C for subsequent procedures.

To investigate the endolithic microbial communities, rock specimens were first washed three times with distilled water. The specimens were then surface sterilized in a 1% sodium hypochlorite solution for 10 min. After surface sterilization, the samples were rinsed five times with sterile distilled water and an additional two to three times with sterile deionized water. The rocks were then placed on sterile tissue paper to air-dry in a class II biosafety cabinet. Once dried, the rocks were crushed into powder using a geologist’s hammer.

Bulk soil samples were aseptically collected from soil surrounding the base of sandstone and limestone substrates at the rock–soil interface (within approximately 0–20 cm) for comparison with lithic microbial communities. The soil samples were preserved in a sterile 50 mL centrifuge tube containing DNA/RNA Shield Reagent (Zymo Research, Irvine, CA).

### 2.2 Genomic DNA extraction and sequencing

Total microbial genomic DNA from epilithic communities on limestone and sandstone substrates was extracted using the DNeasy^®^ PowerSoil^®^ Pro Kit (QIAGEN, Germany), following the manufacturer’s instructions. Genomic DNA was extracted with the DNeasy^®^ PowerMax^®^ Soil Kit (QIAGEN, Germany) for endolithic community within limestone, according to the manufacturer’s guidelines. Attempts to extract genomic DNA from endolithic community within sandstone were unsuccessful due to insufficient DNA yield for downstream sequencing. Genomic DNA from soil samples surrounding the sandstone and limestone was extracted using the DNeasy^®^ PowerSoil^®^ Pro Kit (QIAGEN, Germany). Bacterial and fungal communities were analyzed by targeting the 16S rRNA V3-V4 region (338F: 5′-ACTCCTACGGGAGGCAGCA-3′ and 806R: 5′-GGACTACHVGGGTWTCTAAT-3′) (Caporaso et al., 2011; Huse et al., 2008) and the ITS2 region (ITS3: 5′-GCATCGATGAAGAACGCAGC-3′ and ITS4: 5′-TCCTCCGCTTATTGATATGC-3′), respectively (White et al., 1990). Sequencing was conducted using the Illumina NovaSeq SP (PE250) platform at Biomarker Technologies (Kowloon, Hong Kong). The datasets were deposited in the Sequence Read Archive (SRA) of NCBI under the BioProject accession PRJNA1247724 and BioSample accessions SAMN47836674-769.

### 2.3 Microbial community analysis

Microbial community analysis was performed using QIIME2 version 2023.7 (Bolyen et al., 2019). For bacterial community analysis, primers were removed using Cutadapt (Martin, 2011). Chimeric reads were filtered, and amplicon sequence variants (ASVs) were identified using DADA2 (Callahan et al., 2016). Sequences that passed the quality checks for bacteria were retained after quality filtering. Reference sequences (Silva 138 SSURef NR99 full-length) from the Silva 138 database (Quast et al., 2012; Robeson II et al., 2021) were extracted using the primers 338F (5′-ACTCCTACGGGAGGCAGCA-3′) and 806R (5′-GGACTACHVGGGTWTCTAAT-3′) to generate a dataset specifically covering the V3-V4 region. These extracted sequences were used to train a Naïve Bayes classifier, which was subsequently employed to assign taxonomy to the bacterial sequences.

For fungal community analysis, ITSxpress version 2.0.0 was used to remove conserved sequences flanking the ITS2 region (Rivers et al., 2018), and the denoising step with DADA2 was performed to generate ASVs. These ASVs were assigned using a Naïve Bayes classifier trained on the reference sequences from UNITE database (version 9.0, release date 2023-07-18), clustered at 97% similarity (3% distance) into species hypotheses (SHs) (Abarenkov et al., 2023a; Abarenkov et al., 2023b). The singletons, doubletons, Mitochondria, Chloroplasts, and unassigned taxa were filtered using the R package phyloseq (version 1.46.0) (McMurdie and Holmes, 2012). Rarefaction curves for the 16S rRNA V3-V4 region and the ITS2 region were analyzed, and the data were rarefied to the saturated ASV count to ensure an unbiased assessment of microbial diversity. Bacterial and fungal relative abundances were then visualized as pie charts and stacked bar plots using the R package ggplot2 (version 3.5.1) (Wickham, 2016) in RStudio (version 4.3.3) (R Core Team, 2023).

### 2.4 Statistical analyses

Alpha diversity indices, including Shannon diversity and observed features, were calculated using the R package phyloseq (version 1.46.0) (McMurdie and Holmes, 2012) to evaluate species richness and evenness within microbial communities in sandstone and limestone. These results were visualized using boxplots generated with the R package ggplot2 (version 3.5.1) (Wickham, 2016). Statistical differences in observed ASV were tested with the Kruskal-Wallis test, followed by pairwise Wilcoxon tests with Benjamini–Hochberg correction. Shannon’s diversity was analyzed using ANOVA with Tukey’s HSD for *post hoc* comparisons.

For beta diversity analysis, principal coordinates analysis (PCoA) was performed with the phyloseq package to evaluate the dissimilarity of lithic microbial communities based on Bray-Curtis distance. The statistical significance of these dissimilarities was tested using permutational multivariate analysis of variance (PERMANOVA) via the adonis2 function in the R package vegan (version 2.6–8) (Oksanen et al., 2024).

Differential abundance analysis was conducted using Linear discriminant analysis Effect Size (LEfSe) (Segata et al., 2011) with the R package MicrobiomeMarker (version 1.8.0) (Cao et al., 2022) to identify significantly different features. Functional prediction was performed using the R package microeco (version 1.12.0) (Liu et al., 2020), using the FAPROTAX (version 1.2.10) prokaryotic trait database (Louca et al., 2016) and the FUNGuild fungal trait database (Nguyen et al., 2016). All analyses and visualizations were conducted in RStudio (version 4.3.3) (R Core Team, 2023).

## 3 Results

### 3.1 Alpha diversity and beta diversity of bacteria and fungi in sandstone and limestone

**Figure 2:**
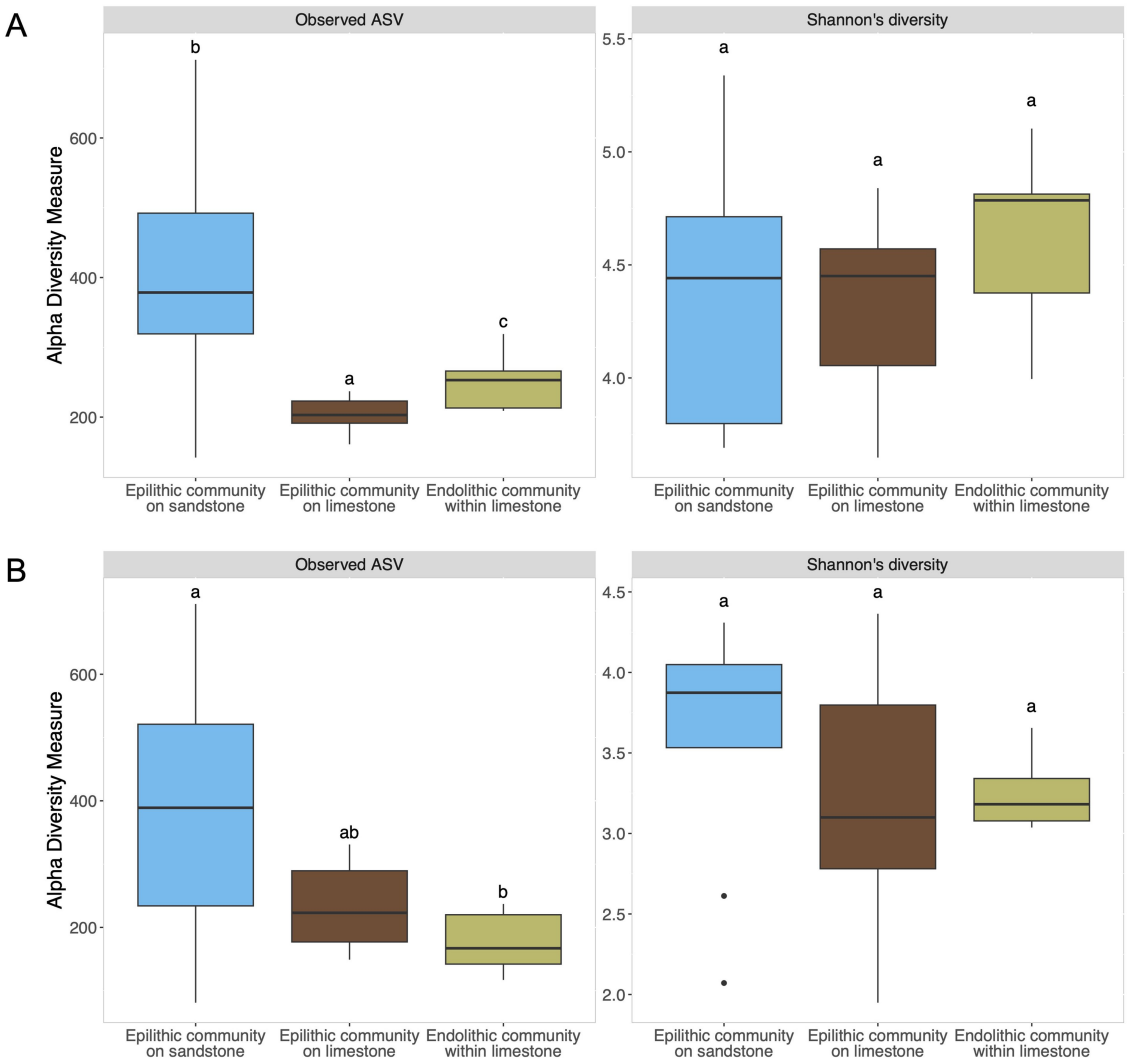
Alpha diversity measurement of bacterial communities **(A)** and fungal communities **(B)** of distinct lithic habitats of sandstone and limestone substrates. The measurement metrics were estimated at amplicon sequence variants (ASVs). Statistical differences in observed ASV were tested with the Kruskal–Wallis test, followed by pairwise Wilcoxon tests with Benjamini–Hochberg correction. Shannon’s diversity was analyzed using ANOVA with Tukey’s HSD for *post hoc* comparisons. Letters depict significant differences (*p* < 0.05) among the habitats. Sample sizes were 8, 10, and 9 for bacterial communities, and 9, 10, and 10 for fungal communities, corresponding to epilithic communities on sandstone, epilithic communities on limestone, and endolithic communities within limestone, respectively.

The microbial communities included bacterial and fungal populations associated with sandstone and limestone substrates. Limestone samples comprised both endolithic and epilithic microbial communities, whereas sandstone samples included only epilithic communities, as DNA extraction from endolithic communities was unsuccessful. For alpha diversity, richness measured by observed features (ASVs) was highest in epilithic bacterial and fungal communities on sandstone (400.00 ± 174.00 and 404.00 ± 212.00, respectively) (Figure 2). Comparatively, the richness of epilithic bacterial community on limestone (202.00 ± 25.8) was lower than that of epilithic bacterial community on sandstone (400.00 ± 174.00). Similarly, epilithic fungal community on limestone (236 ± 68.3) displayed lower richness than that of epilithic fungal community on sandstone (404.00 ± 212.00). The comparison of epilithic bacterial communities between sandstone and limestone showed a statistically significant difference (*p* < 0.05), whereas fungal communities did not show a statistically significant difference (*p* > 0.05) (Figure 2).

When comparing endolithic and epilithic communities associated with limestone, the richness of the endolithic bacterial community (250.00 ± 36.10) was significantly higher than that of the epilithic bacterial community (202.00 ± 25.8) (*p* < 0.05). Although the Shannon diversity index did not differ significantly among habitats across both substrates (*p* > 0.05), the highest values were observed in endolithic bacterial communities within limestone (4.61 ± 0.342). Comparable values were found in epilithic bacterial communities on limestone (4.33 ± 0.373) and sandstone (4.33 ± 0.609) (Figure 2A). In contrast, the richness of endolithic fungal communities within limestone (176.00 ± 45.6) was lower than that of epilithic fungal communities on limestone (236.00 ± 68.3), although the difference was not statistically significant (*p* > 0.05). Similarly, the Shannon diversity index did not differ significantly among habitats across both substrates (*p* > 0.05). The highest values were observed in epilithic fungal communities on sandstone (3.59 ± 0.776), followed by endolithic fungal communities within limestone (3.24 ± 0.206) and epilithic fungal communities on limestone (3.21 ± 0.726) (Figure 2B).

**Figure 3:**
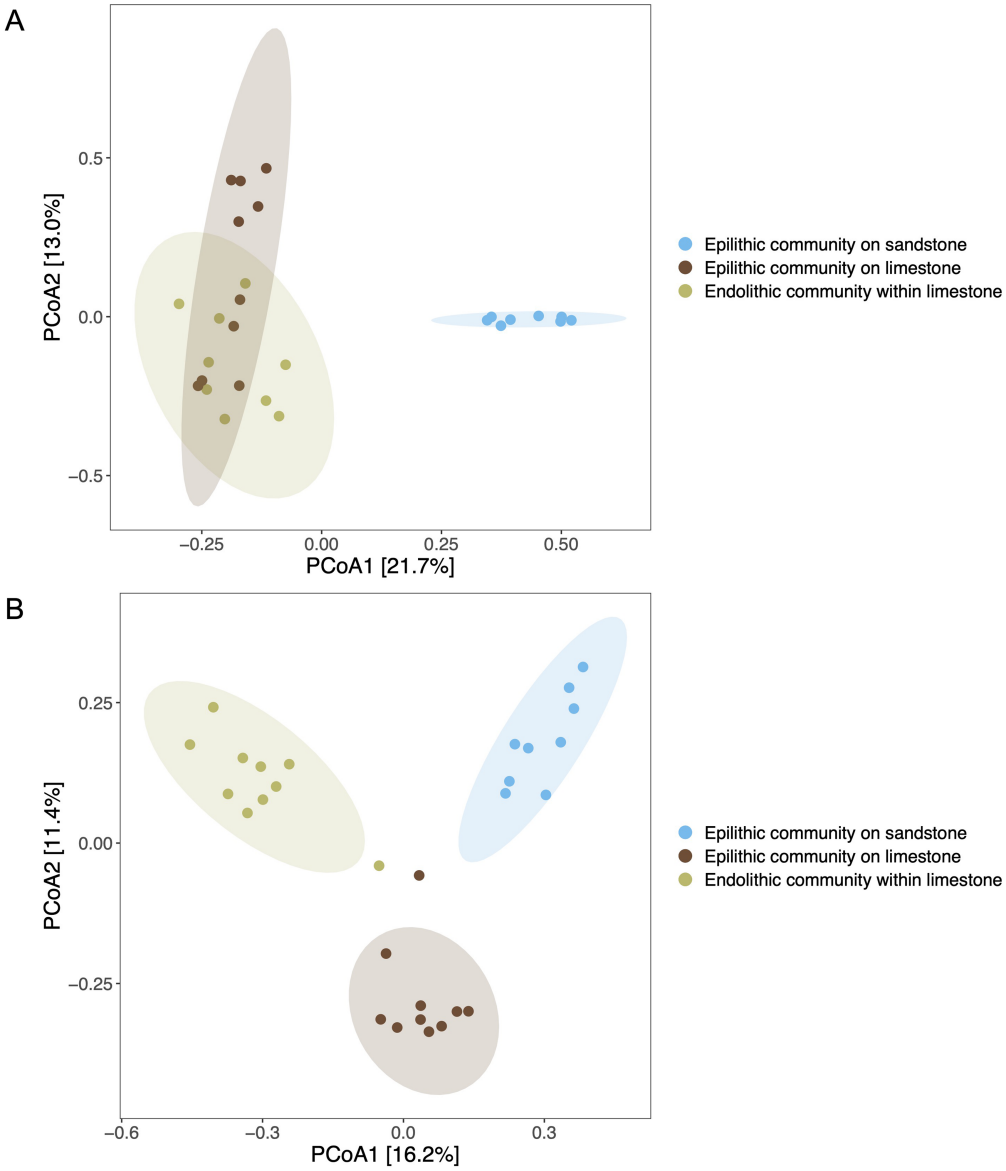
Principal coordinates analysis (PCoA) plot of bacterial communities **(A)** and fungal communities **(B)** from distinct lithic habitats of sandstone and limestone substrates based on Bray-Curtis distance, demonstrating the dissimilarity of microbial communities across habitats. The analysis was conducted on amplicon sequence variants (ASVs). The significant differences were carried out using PERMANOVA with 999 permutations. The ellipse ovals represent 95% confidence ellipses around the group centroids. Percentages on each axis indicate the proportion of variation in community composition explained by the corresponding principal coordinate.

Beta diversity analysis further highlighted these distinctions (Figure 3 and Supplementary Figure S1). Epilithic bacterial community on limestone showed significant differences in bacterial community structure compared to epilithic bacterial community on sandstone, based on Bray–Curtis dissimilarity (*p* = 0.001, *R*^2^ = 0.271), as shown in the PCoA plot (Figure 3A). The higher *R*^2^ value indicated that substantial proportions of variation (27.1%) were associated with differences between sandstone and limestone substrates. In contrast, the comparison between epilithic and endolithic bacterial communities associated with limestone revealed a lower *R*^2^ value of 0.158 (*p* = 0.001).

For fungal communities, the dissimilarity between epilithic communities on sandstone and limestone (*p* = 0.001, *R*^2^ = 0.180) was observed in the PCoA plot (Figure 3B). Comparisons between epilithic and endolithic fungal communities associated with limestone showed an *R*^2^ value of 0.198 (*p* = 0.001).

The soils surrounding sandstone and limestone rocks were collected and analyzed using beta diversity metric to elucidate and compare microbial community differences between the surrounding soils and lithic habitats. Beta diversity analysis revealed significant differences in microbial communities between soils and lithic habitats. For the bacterial community, soil community surrounding sandstone differed from epilithic community on sandstone (*p* = 0.001, *R*^2^ = 0.293). Similarly, soil community surrounding limestone differed from epilithic and endolithic communities associated with limestone (*p* = 0.001, *R*^2^ = 0.387 and 0.388, respectively).

Significant dissimilarity in fungal communities was observed between soils and rocks (sandstone and limestone) (PERMANOVA, *p* = 0.001). The variation explained between soil community surrounding sandstone and epilithic community on sandstone was 21.2% (*R*^2^ = 0.212). A higher proportion of variation (25.6%) was observed between soil community surrounding limestone and endolithic community within limestone, whereas a lower variation (15.1%) was observed between soil community surrounding limestone and epilithic community on limestone.

### 3.2 Lithic microbial community structure

**Figure 4:**
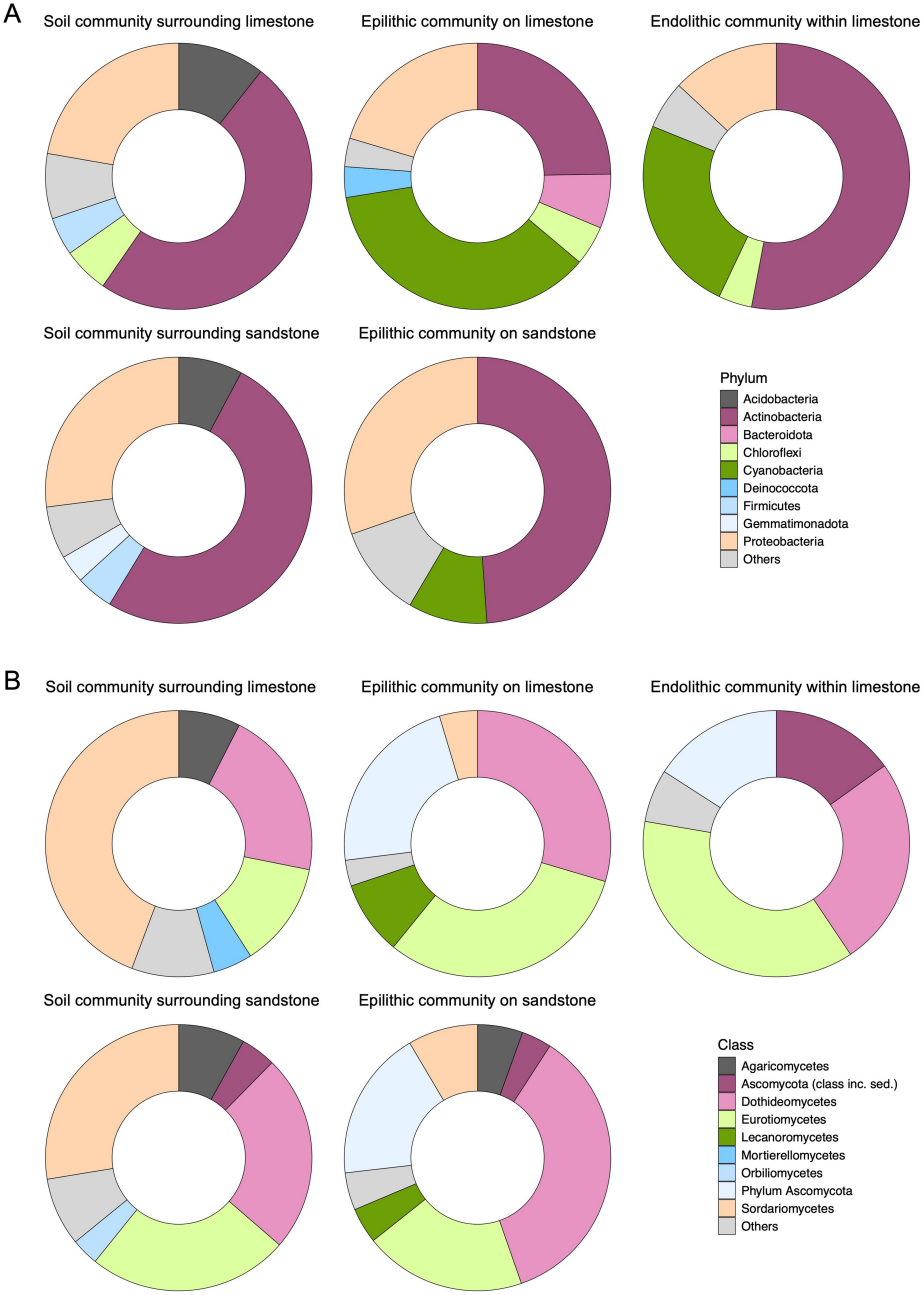
Bacterial relative abundance **(A)** and fungal relative abundance **(B)** of soils surrounding the rocks and distinct lithic substrates of sandstone and limestone. Bacterial phyla and fungal classes with a total relative abundance below 3% were grouped under ‘Others’. Sample sizes were 10, 8, 10, 10, and 9 for bacterial communities, and 10, 9, 10, 10, and 10 for fungal communities, corresponding to soil communities surrounding sandstone, epilithic communities on sandstone, soil communities surrounding limestone, epilithic communities on limestone, endolithic communities within limestone, respectively.

The relative abundances of lithic microbial communities revealed notable differences in community structure across the various lithic habitats. The dominant bacterial phyla were Actinobacteria (18.40–80.40%), Proteobacteria (5.37–53.60%), and Cyanobacteria (4.57–53.39%) across all habitats. Cyanobacteria were more abundant in epilithic and endolithic communities associated with limestone (25.14–53.38% and 15.12–43.06%, respectively) compared to epilithic community on sandstone (4.57–25.14%). Other phyla, such as Chloroflexi (3.02–9.10%) and Deinococcota, were detected across lithic habitats at relatively low abundance. Deinococcota ranged from 0.03 to 5.28% in epilithic communities on sandstone, 0.46 to 9.65% on limestone, and 0.03 to 2.4% in endolithic communities within limestone. (Figure 4A, Supplementary Figure S2).

Soil bacterial communities surrounding sandstone had a distinct structure from epilithic community on sandstone. Similarly, soil communities surrounding limestone showed different community structures than those in limestone lithic habitats. The dominant phyla in soil communities surrounding sandstone and limestone were Actinobacteria (46.53–53.95% and 41.42–59.52%, respectively), Acidobacteria (6.05–9.69% and 5.98–15.62%, respectively), and Proteobacteria (23.11–33.07% and 19.58–24.54%, respectively). When comparing soil and lithic communities, Cyanobacteria accounted for less than 3% of the soil communities under investigation (Figure 4A, Supplementary Figure S2).

Compared to the bacterial community, the relative abundance of Archaea was much lower, with the highest abundance observed at ~0.2% (Supplementary Figure S3). The archaeal classes detected in the lithic habitats included Halobacteria, Nanoarchaeia, Odinarchaeia, and Thermoplasmata.

In fungal communities, Ascomycota and Basidiomycota were the predominant phyla. Ascomycota accounted for over 90% of the fungal community across all lithic habitats, while Basidiomycota contributed less than 10% (Supplementary Figure S4). At the class level, Dothideomycetes (6.24–67.77%) and Eurotiomycetes (3.58–60.78%) were prevalent across all soil and lithic habitats. Notably, Lecanoromycetes were present at relatively low proportions across lithic habitats compared to the dominant classes, reaching up to 14.87% in epilithic communities on sandstone, 4.59% in epilithic communities on limestone, and 9.54% in endolithic communities within limestone (Figure 4B; Supplementary Figure S5). One limestone epilithic sample exhibited an anomalously high relative abundance (78.46%). The structure of fungal communities in soils differed from those associated with lithic habitats. Sordariomycetes were more abundant in soil communities surrounding both sandstone and limestone (19.44–75.07%) compared to all lithic communities (3.04–15.18%) (Figure 4B, Supplementary Figure S5).

### 3.3 Differential abundance analysis of lithic microbial communities

**Figure 5:**
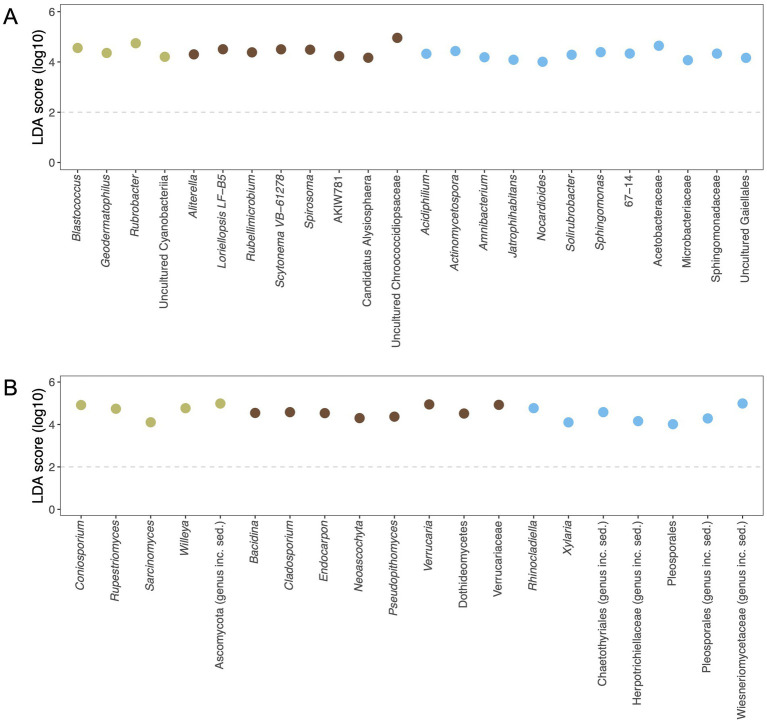
Differential abundance analysis based on LEfSe, illustrating the unique microbial taxa in lithic habitats. **(A)** Bacterial community. **(B)** Fungal community. The analysis was identified at the genus level. The enriched microbial taxa were plotted at the size effect of linear discriminant analysis (LDA) > 4. The dashed line indicates a default LDA. Colored circles respected to microbial taxa indicate their occurrence in different lithic communities: endolithic communities within limestone (olive-green), epilithic communities on limestone (brown), and epilithic communities on sandstone (blue). Differential taxa were derived from the calculation by the Kruskal-Wallis test (*p* < 0.05) and estimation through the Wilcoxon rank-sum test (*p* < 0.05).

Several bacteria and fungi exhibited differential abundance across the lithic habitats. The bacterial lineages in limestone (both epilithic and endolithic habitats) were enriched in phototrophic microorganisms. In bacterial community associated with limestone, uncultured members of the class Cyanobacteriia within the phylum Cyanobacteria were identified in endolithic community within limestone, along with radio-resistant bacteria such as *Rubrobacter* and *Geodermatophilus*. Within the epilithic community on limestone, the bacterial community featured a variety of cyanobacterial taxa, including *Aliterella*, *Loriellopsis*, *Scytonema*, and uncultured Chroococcidiopsaceae. In contrast, the epilithic bacterial community on sandstone was primarily composed of heterotrophic bacteria (Figure 5A).

The fungal communities also demonstrated habitat-specific compositions, predominantly comprising rock-inhabiting fungi (RIF). Endolithic community within limestone was characterized by fungal taxa such as *Coniosporium*, *Rupestriomyces*, *Sarcinomyces*, and *Willeya*. Epilithic community on limestone exhibited distinct fungal taxa, including *Verrucaria*, *Endocarpon*, and *Cladosporium*, as well as one lineage of lichenized fungi, *Bacidina*. By comparison, the epilithic fungal community on sandstone was primarily composed of *Rhinocladiella* (Figure 5B).

### 3.4 Functional prediction of bacterial and fungal community

A total of 46 and 26 putative functions were inferred for bacterial and fungal communities across lithic habitats, respectively (Supplementary Tables S1, S2). For fungi, all confidence levels provided by the FUNGuild database (“highly probable,” “probable,” and “possible”) were included to explore the full spectrum of predicted functional diversity (Supplementary Table S3).

**Figure 6:**
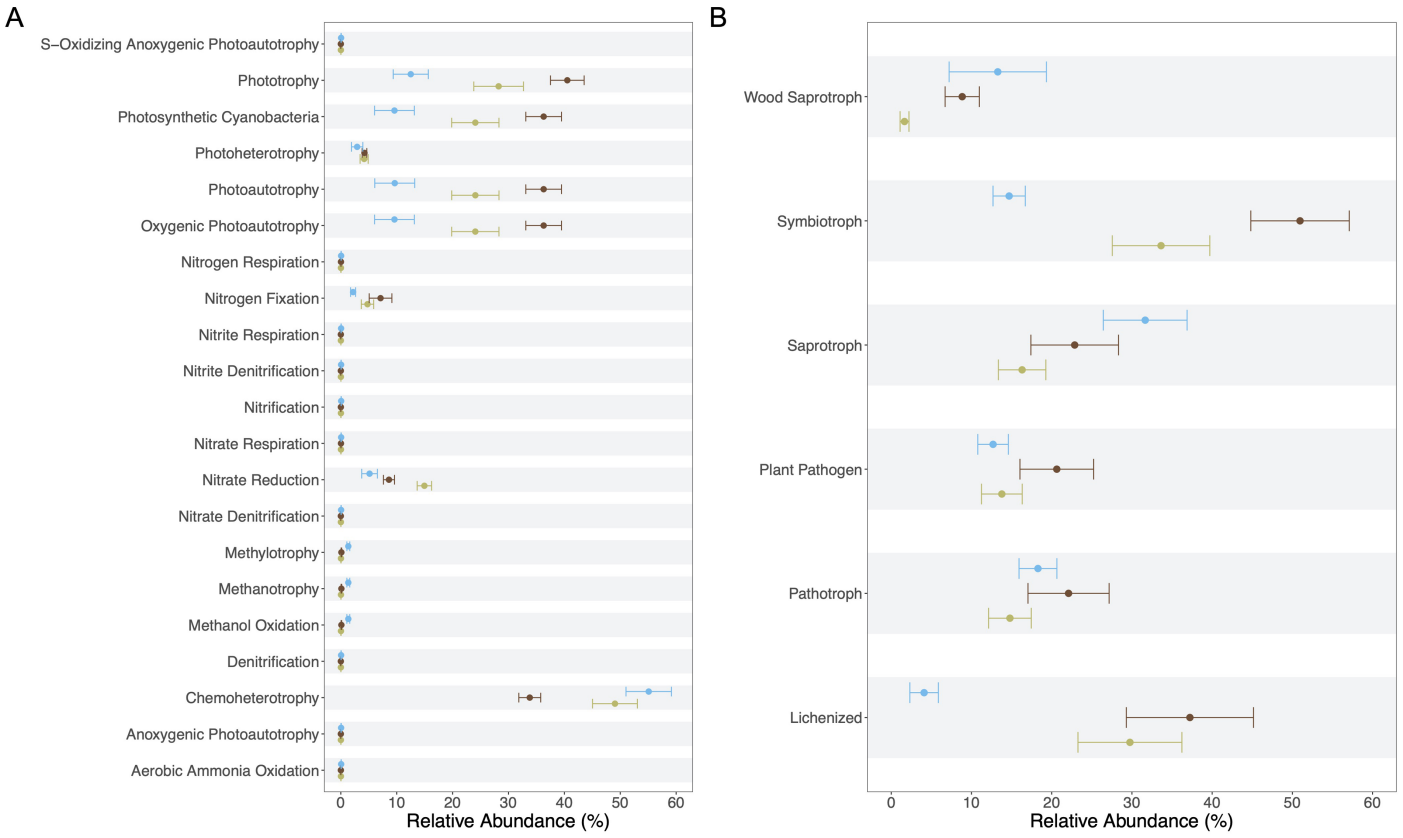
Predicted functional profiles of microbial communities across distinct lithic habitats. **(A)** Bacterial community based on FAPROTAX assignments. **(B)** Fungal community based on FUNGuild predictions. Functions represent putative or inferred ecological roles derived from taxonomic associations rather than direct metabolic evidence. Points represent mean relative abundance, and error bars indicate standard error (SE). Colors denote habitat–substrate associations: endolithic communities within limestone (olive-green), epilithic communities on limestone (brown), and epilithic communities on sandstone (blue).

In the bacterial community, FAPROTAX assignments suggested functions primarily related to carbon and nitrogen cycling. Chemoheterotrophy was the most common predicted function across all habitats. Processes related to methanol oxidation, methanotrophy, and methylotrophy appeared more prominent in epilithic community on sandstone, suggesting distinct carbon-processing capabilities. Light-dependent processes such as phototrophy, photoautotrophy, oxygenic photoautotrophy, and photoheterotrophy were also inferred across all habitats, while anoxygenic photoautotrophy and sulfur-oxidizing anoxygenic photoautotrophy were detected in epilithic community on sandstone. These categories overlap, as FAPROTAX maps taxa to multiple related functions based on the literature (Figure 6A). Predicted nitrogen-related functions included nitrate reduction and nitrogen fixation were prominent in limestone (both epilithic and endolithic habitats). In contrast, epilithic community on sandstone exhibited a broader set of predicted nitrogen functions, including nitrification, denitrification, and aerobic ammonia oxidation.

## 4 Discussion

### 4.1 Core lithobionts and hidden diversity in tropical lithic environments

Our study revealed variations in microbial diversity across lithic habitats based on alpha diversity analysis, suggesting that lithology may influence these differences. In epilithic habitats, both bacterial and fungal diversity tended to be higher on sandstone than on limestone, suggesting that the relatively lower environmental stress at the sandstone site, such as indirect sunlight exposure and higher humidity, may provide more favorable conditions for microbial colonization. This observed distinction in community composition between limestone and sandstone further suggests that substrate characteristics could contribute to shaping lithic microbial communities.

Within limestone, a comparison of epilithic and endolithic niches (separated by only a few millimeters) suggests that endolithic habitats facilitate the colonization by a broader range of bacterial taxa. However, the weaker differentiation in bacterial communities between these niches likely reflects shared environmental factors and close spatial proximity. In contrast, fungal diversity was higher in epilithic than in endolithic habitats, which may reflect the heterotrophic or saprotrophic lifestyles of fungi. These organisms likely rely on organic matter, which is more available at surface environments where phototrophic microorganisms are more abundant than within the rock matrix.

Choe et al. (2021) reported that sandstone and limestone exhibit distinct physical and chemical properties, supporting significant differences in microbial community composition. Additionally, environmental factors, e.g., precipitation, temperature, and vegetation coverage, likely drive lithic microbial diversity at our sampling sites, as Zhao et al. (2023) observed variations in hypolithic bacterial and fungal diversity across distant temperate-climate sites. Furthermore, the distinct bacterial and fungal communities observed among lithic habitats and between lithic and soil environments highlight the influence of ecological niches and substrate characteristics. Consistent with this, Choe et al. (2021) reported substantial differences in microbial communities between rocks and adjacent soils. However, the observed variation in fungal communities suggests that factors beyond the immediate soil–rock interface may also play a role, reflecting the complex interplay among substrate properties, environmental conditions, and broader geographical influences in shaping microbial diversity.

The relative abundance of bacteria in lithic communities from sandstone and limestone in this study showed a consistent pattern, characterized by high proportions of Actinobacteria and Proteobacteria. These findings align with previous studies from cold and dry regions, where Actinobacteria and Proteobacteria were dominant across various rock types (Choe et al., 2018; Choe et al., 2021). Similarly, in warm Mediterranean, cold semi-arid, and hot desert climates, these two phyla were reported to comprise over 70% of stone-associated microbial communities (Louati et al., 2020). Our study extends this pattern to tropical environments, showing that Actinobacteria and Proteobacteria are also dominant in lithic habitats in northern Thailand. These results provide evidence that these two lineages represent core bacterial groups that consistently occupy and thrive in rock-associated microbial niches across diverse climatic regions.

Cyanobacteria were frequently detected in lithic communities across extreme environments, including dry valleys, polar deserts, and hyper-arid deserts (Archer et al., 2017; Choe et al., 2021; Choe et al., 2018; Meslier et al., 2018). In this study, we found the diverse proportions of various cyanobacterial lineages in lithic communities across sandstones and limestones, including *Aliterella* (0.04–22.90%), *Calothrix* (0.03–5.97%), *Chlorogloeopsis* (0.01–0.06%), *Chroococcidiopsis* (0.03–19.36%), *Dapisostemonum* (0.03–0.58%), *Desmonostoc* (0.07–0.25%), *Fischerella* (0.006%), *Leptolyngbya* (0.02–0.04%), *Loriellopsis* (0.19–16.30%), *Nostoc* (0.07–0.49%), *Scytonema* (0.02–15.90%), *Stigonema* (0.26–0.49%), and *Tolypothrix* (0.06–1.25%) (Supplementary Figures S6, S7), suggesting that these prevalent assemblages are potentially responsible for key nutrient production in these tropical lithic habitats. Compared to previous studies, most cyanobacterial lineages identified in this study, except for *Dapisostemonum* and *Desmonostoc*, have previously been reported in lithic habitats (Büdel, 1999; Casero et al., 2021; Kala and Pandey, 2023; Pushkareva et al., 2024; Qu et al., 2020; Schulze-Makuch et al., 2021; Weber et al., 2013), suggesting that these taxa represent typical assemblages in lithic habitats across a wide range of climates. Furthermore, the detection of *Dapisostemonum* and *Desmonostoc* in lithic habitats of the tropical region expands our understanding of cyanobacterial diversity and their colonization potential.

Differential abundance analysis of bacterial communities across lithic habitats revealed distinct taxa, including phototrophic and heterotrophic lineages. Notably, autotrophic members of the phylum Cyanobacteria were consistently enriched, particularly in surface-exposed habitats. LEfSe analysis identified lineage-specific autotrophs that characterize each rock type: members of the class Cyanobacteriia were significantly associated with limestone. These cyanobacteria, especially members of the family Chroococcidiopsidaceae, are well adapted to harsh lithic environments such as desiccation and radiation (Mosca et al., 2019; Wierzchos et al., 2012). Its presence has been widely reported in various lithic substrates, including limestone, sandstone, granite, and quartzite, inhabiting both endolithic and chasmoendolithic niches (Choe et al., 2021; Khomutovska et al., 2021; Pointing et al., 2009). These microhabitats offer refuge in the form of pore spaces and cracks, which *Chroococcidiopsis* can infiltrate, secreting extracellular polymeric substances (EPS) that enhance stress resistance and moisture retention (Choe et al., 2021; Wierzchos et al., 2015). As phototrophs, cyanobacteria such as *Chroococcidiopsis* and *Scytonema* contribute significantly to the production of organic compounds, which serve as vital carbon and energy sources for heterotrophic members of the lithic microbial community. Additionally, *Chroococcidiopsis* has been shown to possess key metabolic pathways involved in phosphonate degradation, urea hydrolysis, and phosphorus recycling (Fernández-Martínez et al., 2021), indicating its role in sustaining nutrient cycles under oligotrophic conditions. Cyanobacteria also contribute to nitrogen inputs through nitrogen fixation, further supporting microbial colonization in nutrient-limited lithic environments (Boison et al., 2004). Our findings support this ecological role, as cyanobacterial taxa were differentially abundant, especially in lithic communities associated with limestone, suggesting that their colonization represents a key survival strategy. Through primary production and nutrient cycling, they likely facilitate the persistence of diverse heterotrophic communities within the rock matrix. Moreover, the presence of potential lichen-forming taxa, including cyanobacteria such as *Scytonema* and *Chroococcidiopsis*, points toward possible symbiotic interactions with fungal partners, which may further enhance ecosystem stability and resilience (Molnár and Farkas, 2010).

Besides oxygenic photoautotrophs, we found anoxygenic photoautotrophs, namely, Chloroflexi, in lithic habitats. Across these tropical lithic habitats, two lineages within the family Chloroflexaceae (FFCH7168, 0.067–0.133% and *Candidatus* Chloroploca, 0.039–0.178%) were detected at low relative abundances in limestone, suggesting a supplementary role in nutrient provision within the lithic microbial community. This is consistent with Choe et al. (2018), who detected this phylum in sandstone and limestone encompassing other rock types, including basalt, granite, and travertine. Chloroflexi were also detected in hypolithic habitats, where they are known to contribute to carbon fixation (Zhao et al., 2023). These findings suggest that phototrophic members of Cyanobacteria and Chloroflexi function as primary producers, facilitating the colonization of heterotrophic microorganisms by enhancing nutrient availability under adverse environmental conditions, including lithic habitats of the tropical climates.

Members of Deinococcota were consistently detected across all samples, although at relatively low abundance. This pattern is consistent with previous reports from extreme climates, where variations in Deinococcota abundance have been observed across different rock types (Choe et al., 2018; Ertekin et al., 2021; Meslier et al., 2018; Zhao et al., 2023). The strong sunlight exposure associated with limestone surfaces may favor the proliferation of Deinococcota, a lineage well known for its exceptional resistance to radiation (Griffiths and Gupta, 2007).

In this study, we consistently observed the presence of heterotrophic genera such as *Rubrobacter* and *Geodermatophilus*, both known for their exceptional resistance to high temperatures, desiccation, and ultraviolet radiation (Genderjahn et al., 2021; Gtari et al., 2012; Pointing et al., 2009), These taxa were differentially abundant in endolithic community within limestone, suggesting that despite their radiotolerance, they may preferentially inhabit rock interiors to mitigate the effects of intense solar radiation in tropical environments. In addition to the well-documented lithobionts, genera such as *Rubellimicrobium* and *Spirosoma* were detected on limestone surfaces (Figure 5), which have not previously been reported in association with lithic environments. *Rubellimicrobium* has been reported to be associated with lichen (Jiang et al., 2019), while *Spirosoma*, a soil-associated genus, has been shown to exhibit resistance to radiation (Cho et al., 2023; Park et al., 2021). These characteristics suggest that both taxa may possess adaptive traits enabling persistence in exposed, nutrient-limited lithic environments. Their presence highlights the potential for tropical rock surfaces to harbor previously unrecognized microbial diversity. These findings underscore the ecological value of tropical lithic habitats as reservoirs of extremotolerant microbes and possibly novel taxa, warranting further exploration to better understand microbial adaptation strategies under nutrient-limited and environmentally stressful conditions.

Archaeal taxa detected in lithic habitats, including Halobacteria, Nanoarchaeia, Thermoplasmata, and Odinarchaeia, exhibit adaptations that enable survival under extreme or nutrient-limited conditions. These groups are known for osmoregulation (Vauclare et al., 2015), membrane and protein stability (Smith et al., 1973), and metabolic versatility supporting carbon, nitrogen, and sulfur cycling (Liu et al., 2021; Macleod et al., 2019; Straková et al., 2025). The detection of Asgard archaea (Odinarchaeia) represents the first report of this lineage in a lithic habitat, emphasizing their ecological adaptability and potential roles in biogeochemical processes within rock environments.

Ascomycota has been reported as the dominant fungal phylum across various rock types, followed by Basidiomycota (Choe et al., 2021; Choe et al., 2018; Coleine et al., 2021a). Lithic fungal communities are often dominated by Ascomycota, particularly RIF, likely reflecting their adaptation to nutrient-poor and/or dry environments (Liu et al., 2022; Louati et al., 2020; Su et al., 2015). These previous findings are consistent with our study of two rock types. We observed Dothideomycetes, Eurotiomycetes, and Lecanoromycetes, which aligns with earlier reports identifying these groups as common constituents of fungal communities in lithic environments. Notably, Lecanoromycetes and Eurotiomycetes have been shown to have significantly higher relative abundances in lithic niches than in edaphic (soil) habitats (Choe et al., 2021), suggesting a strong ecological preference for rock surfaces across diverse climates.

In this study, rock-inhabiting fungi (RIF) and lichenized fungi were prominent components of the fungal assemblages in tropical lithic habitats. Previous work across contrasting climates has identified lichen-forming genera such as *Verrucaria* and *Polyblastia* as important in limestone, and *Atla*, *Porpidia*, and *Candelariella* in sandstone, indicating some consistency in fungal associations with specific rock types (Choe et al., 2021). Many prevalent fungal lineages in lithic habitats are lichenized fungi capable of forming symbiotic associations with photosynthetic partners (Grube and Wedin, 2016). In addition, Dothideomycetes and Eurotiomycetes include RIF, particularly micro-colonial fungi or black fungi, characterized by melanized cell walls (Coleine et al., 2021b). This pigmentation is associated with resistance to desiccation and ultraviolet radiation, potentially contributing to the persistence of microbial communities under environmental stress. Lichenized fungi may also contribute to mineral weathering through processes such as disaggregation, hydration, dissolution, and secondary mineral formation (Choe et al., 2018; De los Ríos et al., 2005). These findings suggest that fungal communities, alongside bacteria, play important roles in nutrient dynamics within lithic ecosystems, rather than phototrophic bacteria alone.

Notably, recent studies in northern Thailand have identified novel RIF from sandstone, including the new genus *Petriomyces* (*Pe. obovoidisporus*) and several new species of *Cladophialophora* and *Exophiala* (Thitla et al., 2022, 2023, 2024). These discoveries highlight the underexplored diversity of fungi in tropical lithic environments and the need for further ecological investigation.

### 4.2 Lithic habitats as self-sustaining microbial ecosystems

Metabolic functions related to light-dependent energy acquisition, including both oxygenic and anoxygenic photosynthesis, were detected in lithic bacterial communities, particularly in limestone. These functions were less pronounced in sandstone, possibly due to reduced light availability at the sampling sites, which were partially shaded by vegetation cover. Lower light exposure may constrain the proliferation of phototrophic microorganisms, thereby reducing the overall abundance of light-dependent metabolic functions. Although the homogeneous pore structure of sandstone has been shown to support stratified microbial communities along vertical light and nutrient gradients, creating distinct microenvironments for different functional groups (Cockell et al., 2002), external environmental factors such as shading may override this potential by limiting light input. In contrast, limestone may further facilitate photoautotrophy by supplying inorganic carbon (carbonate and bicarbonate) through mineral dissolution, which can serve as an additional substrate for photosynthesis alongside atmospheric CO₂ uptake. Indeed, microbial carbonate precipitation and the biological cycling of inorganic carbon in carbonate rocks are well documented, and cyanobacteria are known to play key roles in structuring carbonate substrates and mediating carbonate chemistry (Schneider and Le Campion-Alsumard, 1999). This pattern is consistent with observations from lithic microbial communities on cultural stone monuments, where phototrophic taxa were positively associated with light and humidity (Li et al., 2016).

Our findings suggest that free-living phototrophs and lichenized symbionts could serve as primary producers, providing essential carbon and energy sources supporting a diverse range of heterotrophic organisms within the lithic microbial ecosystem. These producer-consumer interactions contribute to the system’s functional integrity and facilitate microbial life’s persistence in physically harsh and oligotrophic conditions.

Nitrogen cycling in lithic habitats also appears to be functionally complete, beginning with the fixation of atmospheric nitrogen. Members of *Chroococcidiopsis*, a dominant cyanobacterial lineage in lithic communities, are known diazotrophs capable of fixing atmospheric nitrogen into biologically available ammonia (Boison et al., 2004; Fernández-Martínez et al., 2021). This process initiates the nitrogen cycle within the lithic environment, particularly in nutrient-poor niches where exogenous nitrogen sources are limited.

Following the release of ammonia from organic nitrogen through decomposition, the next step in the cycle involves ammonia oxidation, a key process performed by nitrite-producing lineages such as MND1, mle1-7, and Ellin6067 (Nitrosomonadaceae). These taxa, detected in our samples (Supplementary Figure S7), are known to encode genes for ammonia monooxygenase and related pathways, contributing to the conversion of ammonia into nitrite (Aguilar-Rangel et al., 2024). Additionally, SC-I-84 has been identified as a dominant genus in soils subjected to high nitrogen inputs (Yang et al., 2021), suggesting that it may contribute to nitrogen cycling in these tropical lithic habitats. Direct evidence linking IS-44 to nitrogen cycling in lithic habitats remains limited. However, as a member of the family Nitrosomonadaceae, which is known for lithoautotrophic ammonia oxidation, IS-44 may possess similar functional capabilities (Jurado et al., 2020), potentially contributing nitrate to the lithic microbial community.

*Nitrospira*, a known nitrite-oxidizing bacterium (Jurado et al., 2020; Swanner and Templeton, 2011), was detected in sandstone and limestone substrates. Despite its relative scarcity (ranging from 0.006 to 0.339% across samples), the consistent presence of *Nitrospira* suggests that it may play a complementary role in oxidizing nitrite to nitrate. Similar observations have been reported in lithic microbial communities on stone monuments, where members of the phylum *Nitrospirae* were detected at low relative abundances (up to ~0.22%) but showed distributions associated with elevated levels of atmospheric pollutants such as NO₂ (Li et al., 2016). This indicates that a full nitrification pathway, albeit at low abundance, is functionally present and contributes to sustaining nitrogen availability in these systems.

The nitrogen cycle may be completed through denitrification, primarily attributed to *Rhodoplanes*, which was detected in lithic substrates. *Rhodoplanes* is capable of reducing nitrate to nitrogen gases (Hiraishi and Ueda, 1994). Given the low-oxygen or anoxic microenvironments often present within rock crevices and biofilms, the presence of this genus suggests that denitrification could be operating even in these presumably nitrogen-poor habitats.

**Figure 7:**
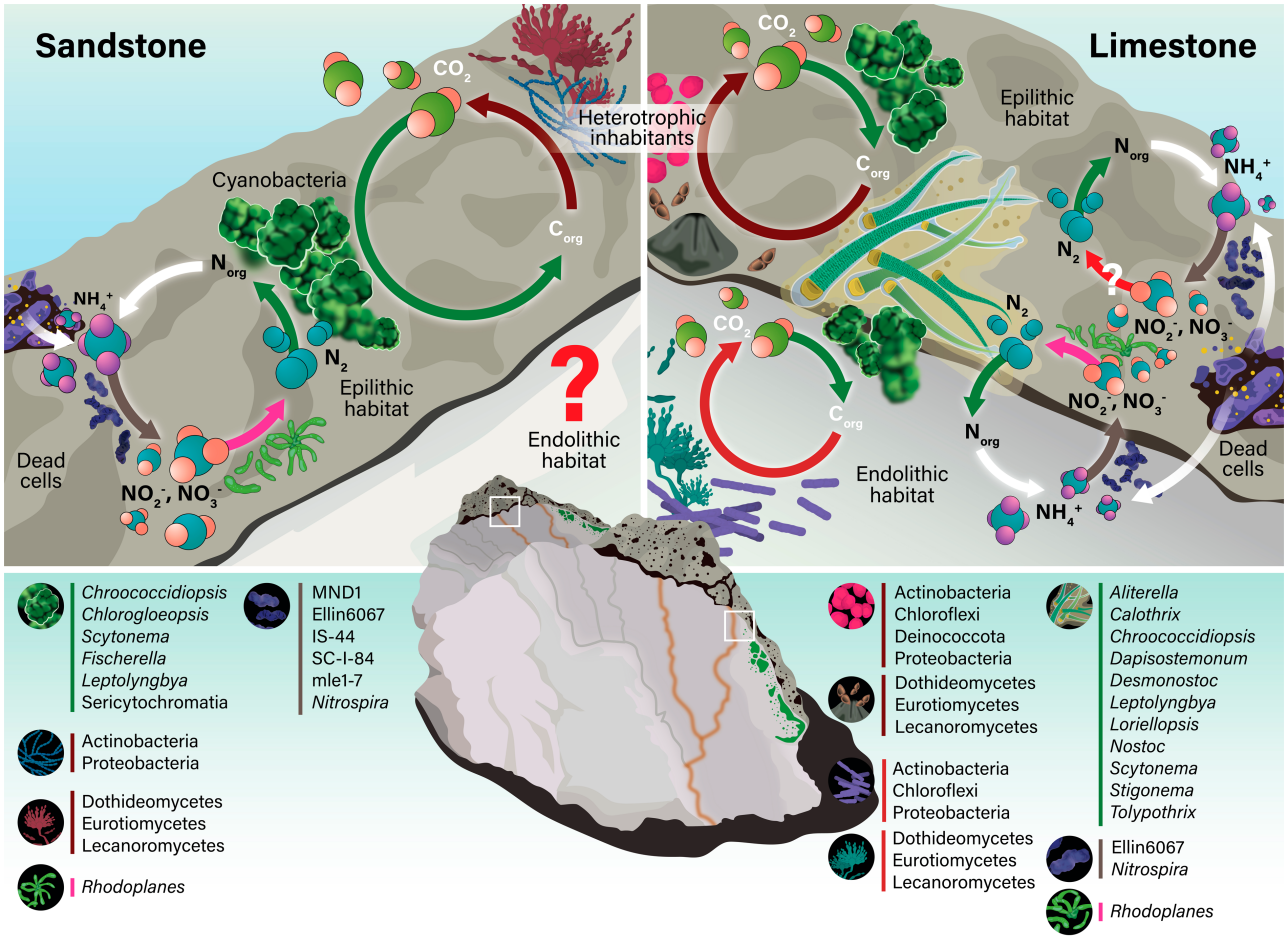
Schematic of biogeochemical cycling mediated by bacterial and fungal communities in epilithic and endolithic niches on sandstone and limestone in northern Thailand. Colored bars adjacent to microbial assemblages correspond to colored arrows representing associated processes. White arrows denote mineralization of organic nitrogen to ammonium via decomposition.

Together, these sequential microbial processes, nitrogen fixation by *Chroococcidiopsis,* ammonia oxidation by Proteobacteria, nitrification by *Nitrospira*, and denitrification by *Rhodoplanes*, demonstrate that the nitrogen cycle operates fully within tropical lithic habitats (Supplementary Figure S7, and Figure 7). A complete nitrogen cycle within these nutrient-poor rock environments further supports the view that lithic habitats function as self-sustaining microbial ecosystems, capable of maintaining internal nutrient dynamics through microbial interactions and metabolic complementarity.

Although no specific phosphorus- or iron-related functions were identified via FAPROTAX annotation, likely due to database limitations (Sansupa et al., 2021), the detection of well-known phosphate-solubilizing bacteria, including *Bacillus*, *Pseudomonas*, *Escherichia-Shigella*, *Enterobacter*, *Serratia*, *Mesorhizobium*, *Acinetobacter*, and *Streptomyces*, suggests active phosphorus mobilization within the lithic system. In addition, *Bacillus*, *Pseudomonas*, *Enterobacter*, and *Streptomyces* are recognized producers of siderophores, facilitating iron acquisition under limiting conditions (Chandran et al., 2021; Wang and Li, 2025) (Supplementary Figure S8). These nutrient mobilization processes are likely critical for sustaining metabolic functionality, enhancing the resilience of lithic microbial assemblages, and underpinning nutrient cycling and broader ecosystem functioning within lithic habitats under tropical conditions.

Overall, while FAPROTAX and FUNGuild highlight putative functions that suggest potential roles of bacteria and fungi in lithic habitats, these predictions remain taxonomically inferred and require validation through direct functional approaches. In the fungal community, the inferred ecological guilds included various saprotrophic, symbiotrophic, and pathogenic lifestyles (Figure 6B). However, these assignments reflect broad taxonomic associations within FUNGuild and may not represent active ecological roles in lithic environments. For example, some taxa annotated as “orchid mycorrhizal,” “leaf saprotroph,” or “animal parasite” likely reflect known associations of these fungi in other habitats rather than active interactions within rocks. To provide transparency, the corresponding taxa associated with these guilds are listed in Supplementary Table S3.

Future research should extend beyond taxonomic characterization to incorporate functional and process-based approaches, including metagenomics, metatranscriptomics, and targeted cultivation, to better resolve the ecological roles and metabolic activities of lithic microbial communities. In addition, investigations of microbe–mineral interactions and expanded spatial and temporal analyses will be essential for understanding the mechanisms governing community assembly and their contributions to biogeochemical processes in lithic environments.

## 5 Conclusion

This study revealed distinct microbial community structures and functional potentials across lithic habitats in sandstone and limestone in northern Thailand. Despite close spatial proximity, significant compositional differences were observed even between epilithic and endolithic communities. Lithic microbiomes, distinct from surrounding soils, were consistently dominated by Actinobacteriota, Proteobacteria, Cyanobacteria, and lichen-forming fungi such as Dothideomycetes, Eurotiomycetes, and Lecanoromycetes. Functional predictions highlighted the coexistence of autotrophs and heterotrophs, with cyanobacteria contributing substantially to carbon and nitrogen fixation. Key metabolic processes, including photosynthesis, heterotrophy, nitrogen cycling (nitrification and denitrification), lichenization, and saprotrophy, were simultaneously present, supporting self-sustaining ecosystems. The detection of phosphate-solubilizing and siderophore-producing genera suggests additional mechanisms for nutrient mobilization. Collectively, these findings position tropical lithic habitats as reservoirs of microbial diversity and functionally integrated ecosystems that contribute to global biogeochemical cycles. They also underscore the resilience of lithic microbial communities under extreme conditions and may guide future studies of tropical lithic ecosystems and their ecological significance.

## Data Availability

The datasets presented in this study can be found in online repositories. The names of the repository/repositories and accession number(s) can be found in the article/Supplementary material.
