# Supplementary material for: Microbial community structure and functional potential of tropical lithic habitats in northern Thailand
Source: Front Microbiol. 2026 May 12;17:1794540. doi: 10.3389/fmicb.2026.1794540 (PMC13201407; doi:10.3389/fmicb.2026.1794540)
Supplement: Supplementary file 1 [file Data_Sheet_1.PDF]

Supplementary Material

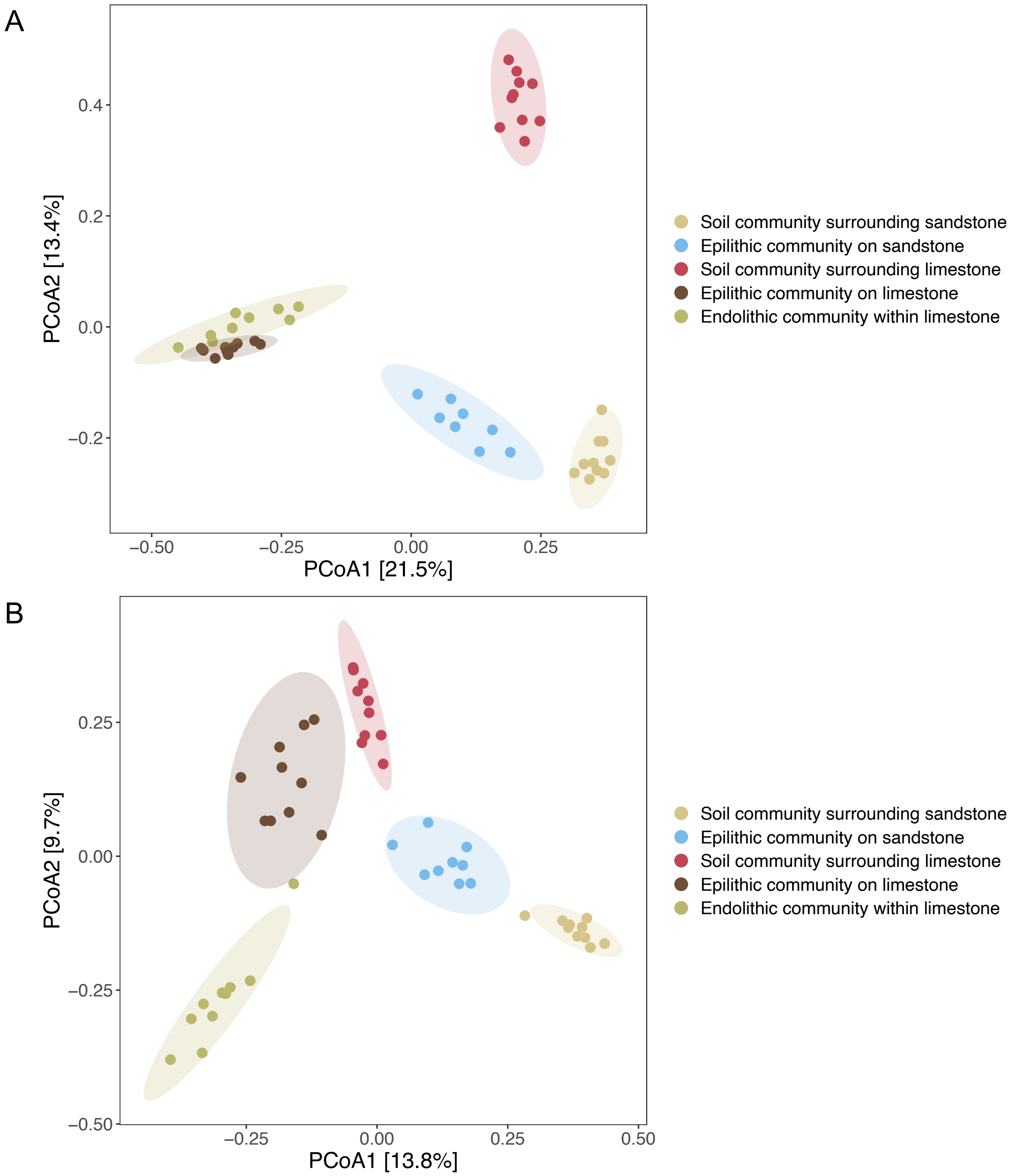

Supplementary Figure 1. Principal coordinates analysis (PCoA) plot based on the Bray-Curtis distance of microbial community from soils surrounding the rocks and distinct lithic substrates

(sandstone and limestone). (A) Bacterial community. (B) Fungal community. The analysis was conducted at amplicon sequence variants (ASVs). The ellipse ovals represent 95% confidence ellipses around the group centroids. Percentages on each axis indicate the proportion of variation in community composition explained by the corresponding principal coordinate.

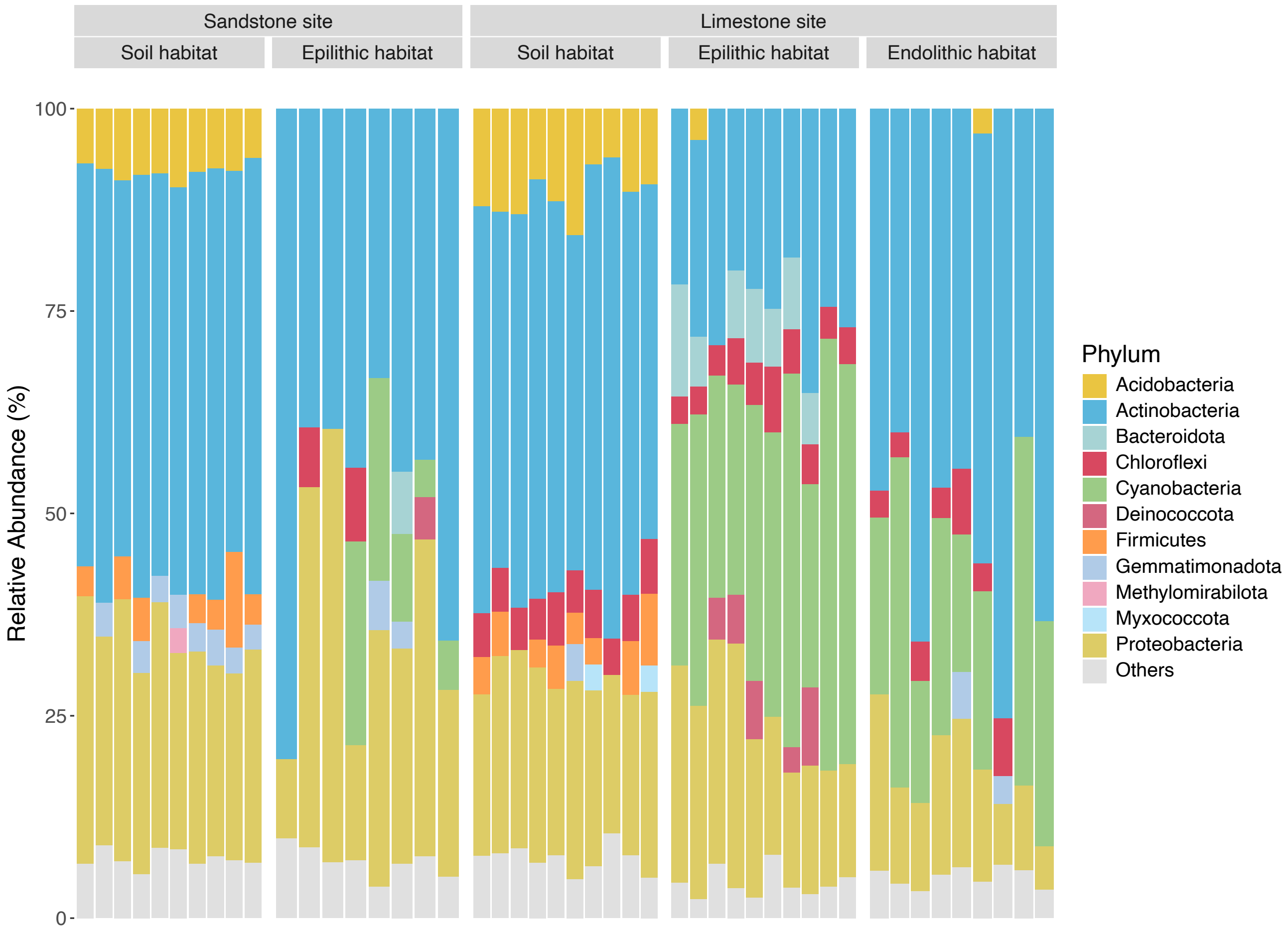

Supplementary Figure 2. Bacterial relative abundance (Phylum level) of soils surrounding the rocks and distinct lithic substrates (sandstone and limestone). Phyla with a relative abundance below 3% were grouped under 'Others'. Each bar referred to each sample.

A

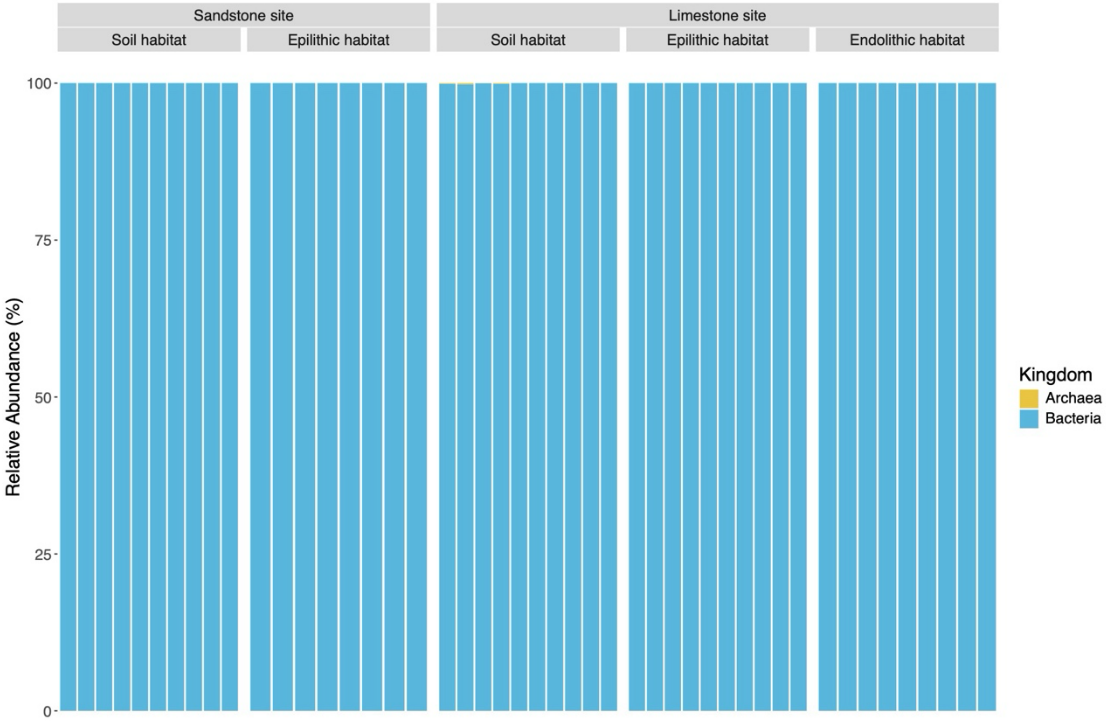
Sandstone site
Limestone site
Soil habitat
Epilithic habitat
Soil habitat
Epilithic habitat
Endolithic habitat
Relative Abundance (%)
100
75
50
25
0
Kingdom
Archaea
Bacteria

B

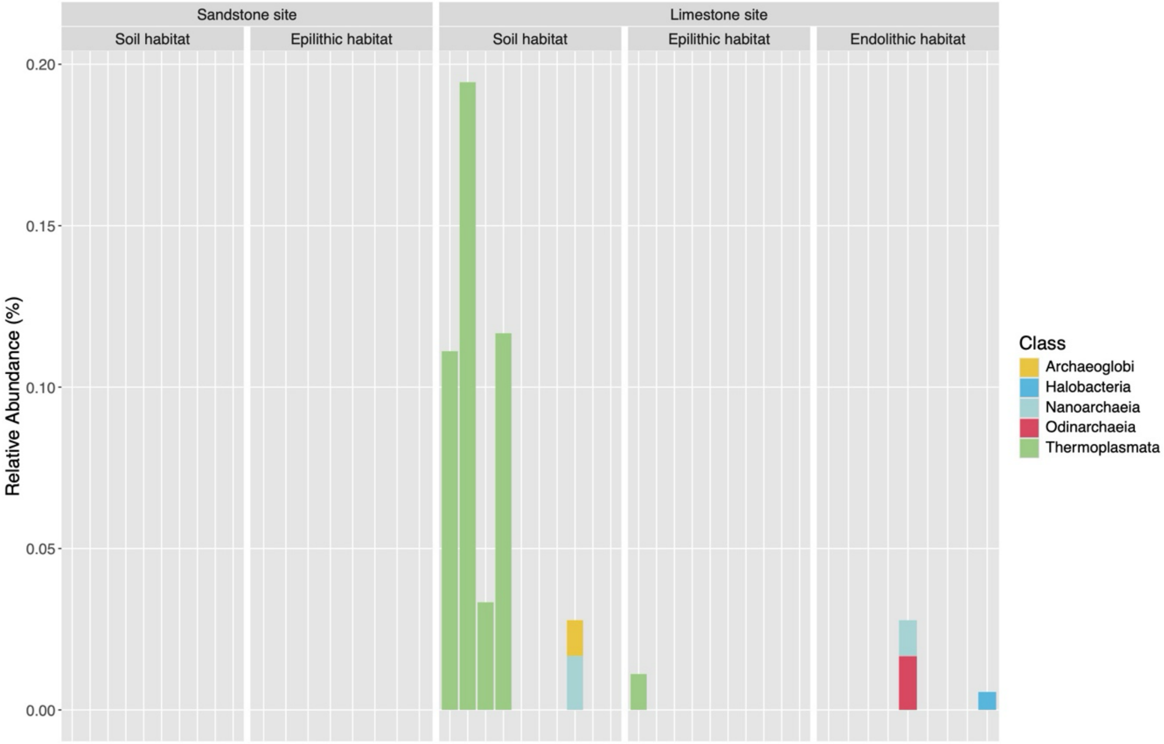
Sandstone site
Limestone site
Soil habitat
Epilithic habitat
Soil habitat
Epilithic habitat
Endolithic habitat
Relative Abundance (%)
0.20
0.15
0.10
0.05
0.00
Class
Archaeoglobi
Halobacteria
Nanoarchaeia
Odinarchaeia
Thermoplasmata

Supplementary Figure 3. Relative abundance of bacteria and archaea in soils surrounding rocks and different lithic substrates (sandstone and limestone (A). The absent bars indicate undetected relative abundance of archaeal classes (B). Each bar represents a sample.

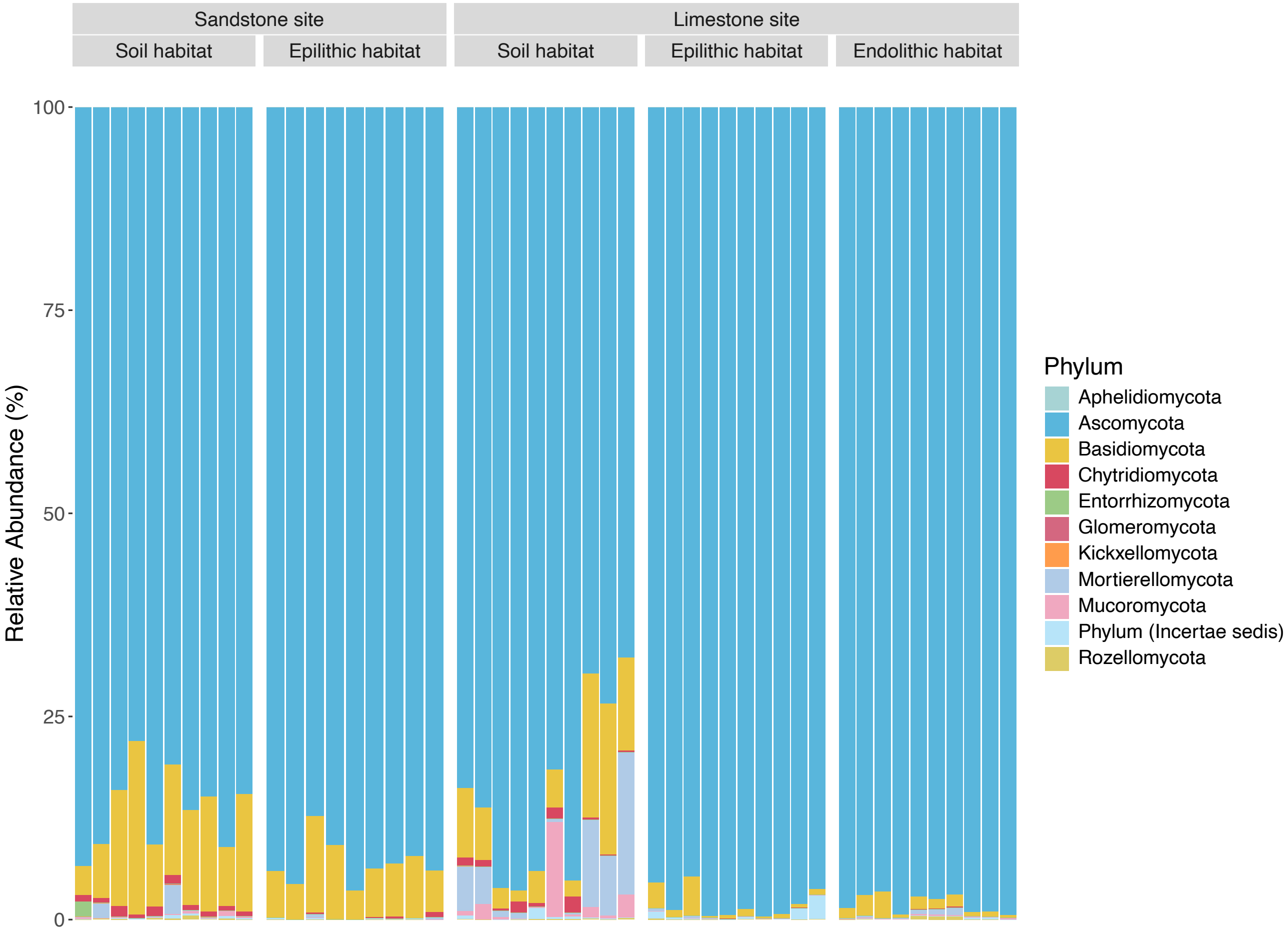

Supplementary Figure 4. Fungal relative abundance (Phylum level) of soils surrounding the rocks and distinct lithic substrates (sandstone and limestone). Each bar referred to each sample.

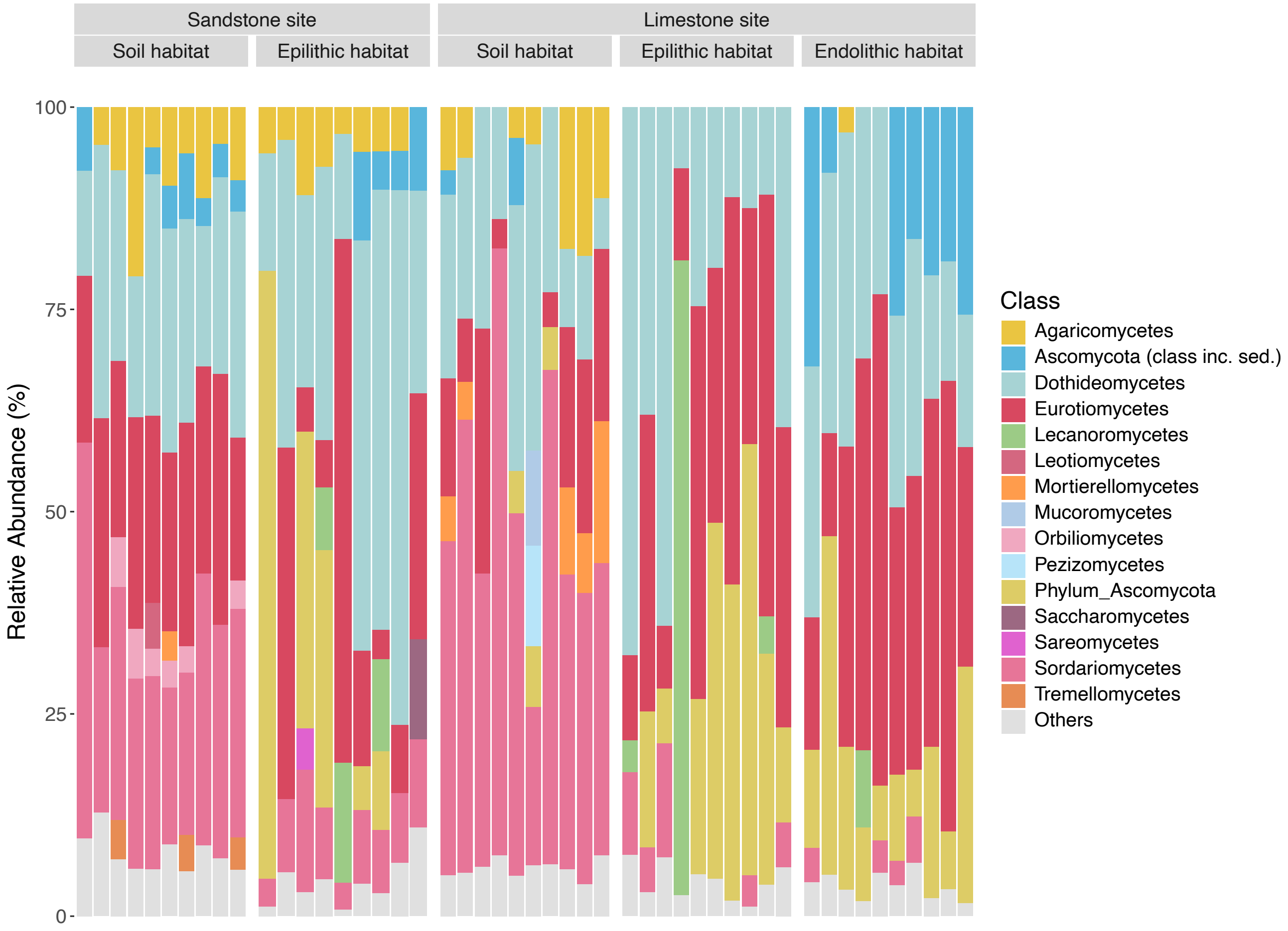

Supplementary Figure 5. Fungal relative abundance (Class level) of soils surrounding the rocks and distinct lithic substrates (sandstone and limestone). Phyla with a relative abundance below 3% were grouped under 'Others'. Each bar referred to each sample.

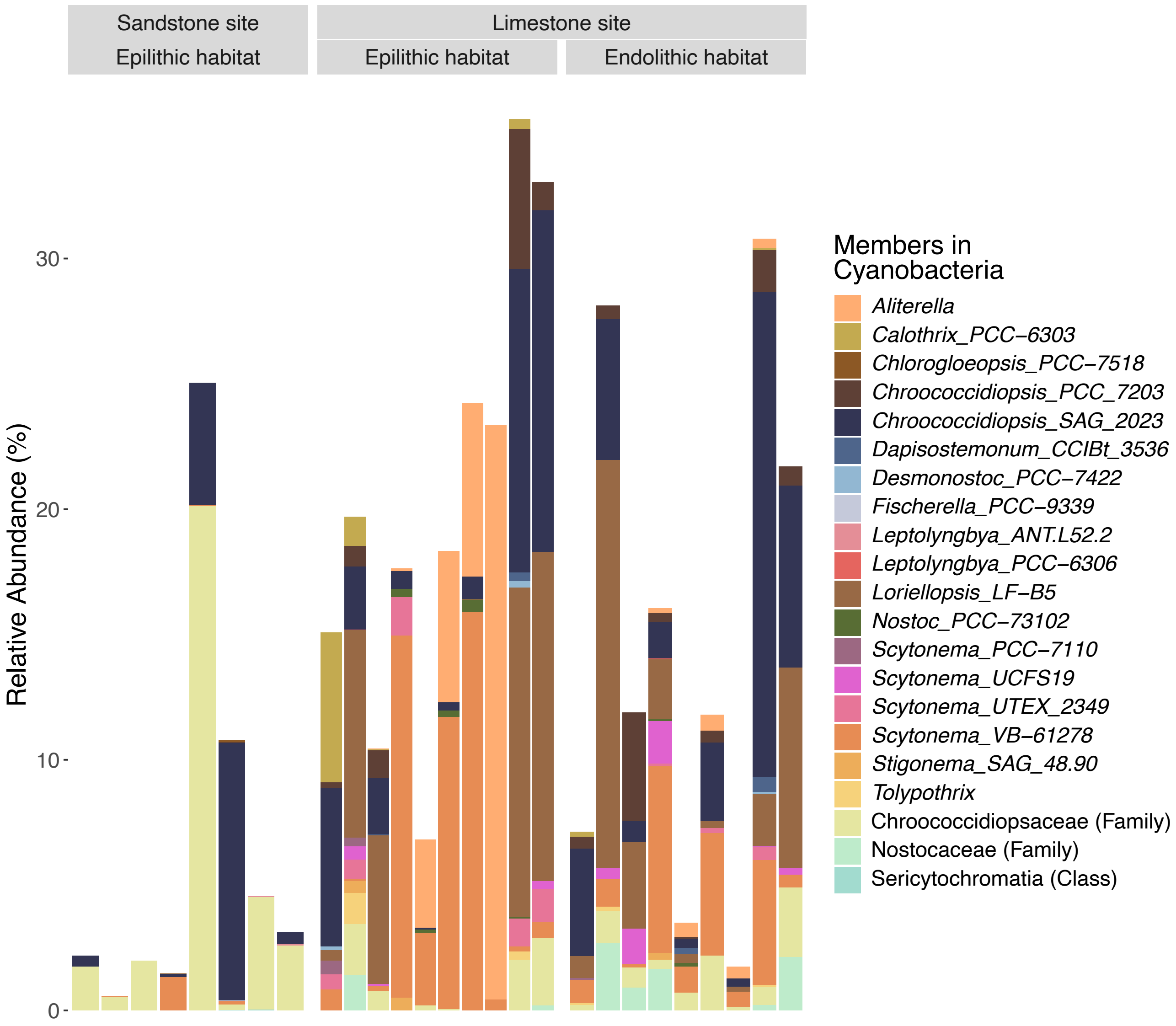

Supplementary Figure 6. Relative abundance of prevalent members in Cyanobacteria at the genus level across lithic habitats. The "uncultured Cyanobacteria" were filtered out to identify relevant cyanobacterial genus members. Each bar referred to each sample.

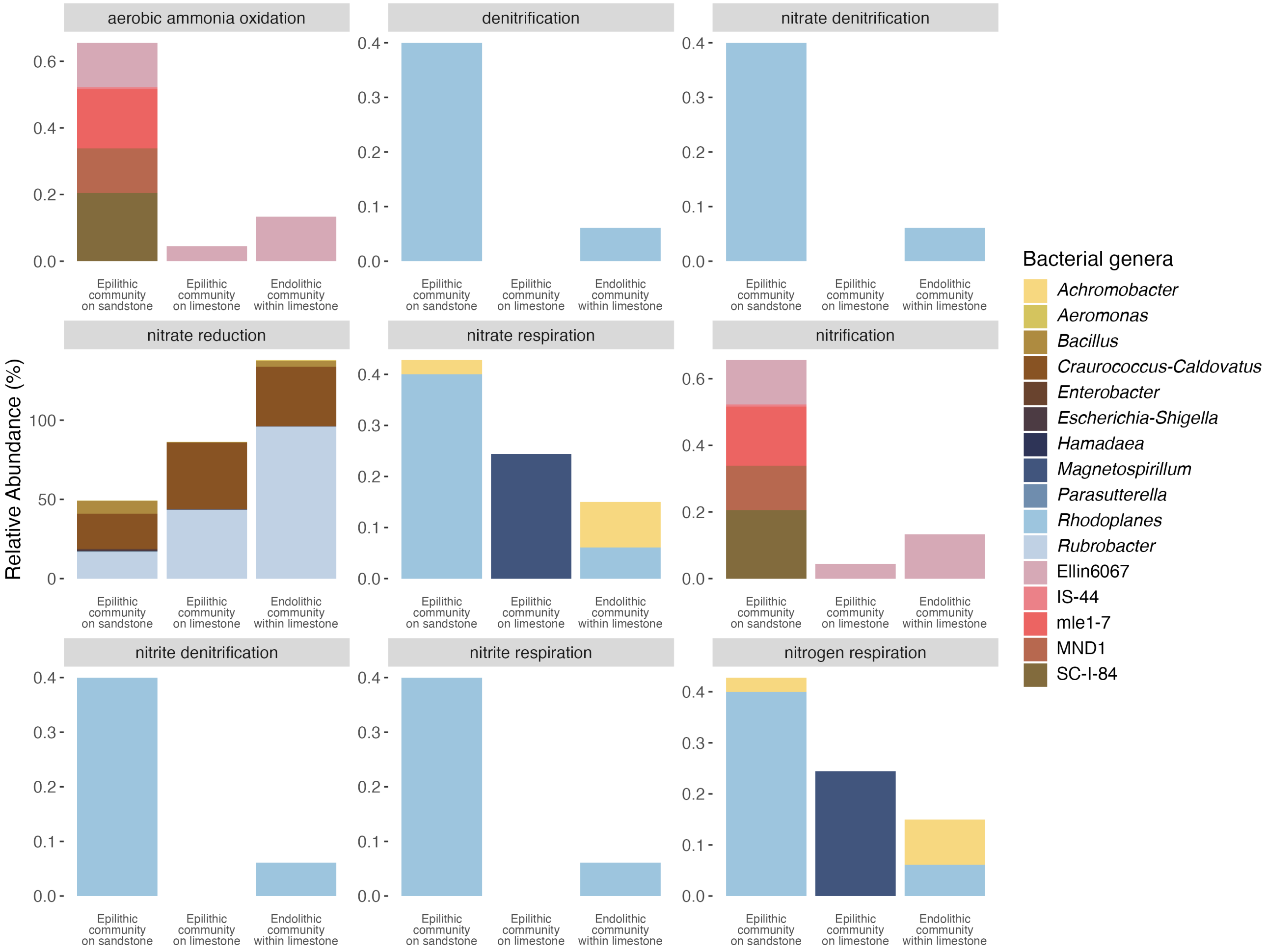

Supplementary Figure 7. Relative abundance of bacterial genera with capabilities of ammonia oxidation, nitrification, and denitrification processes across lithic habitats based on metabolic function prediction using FAPROTAX.

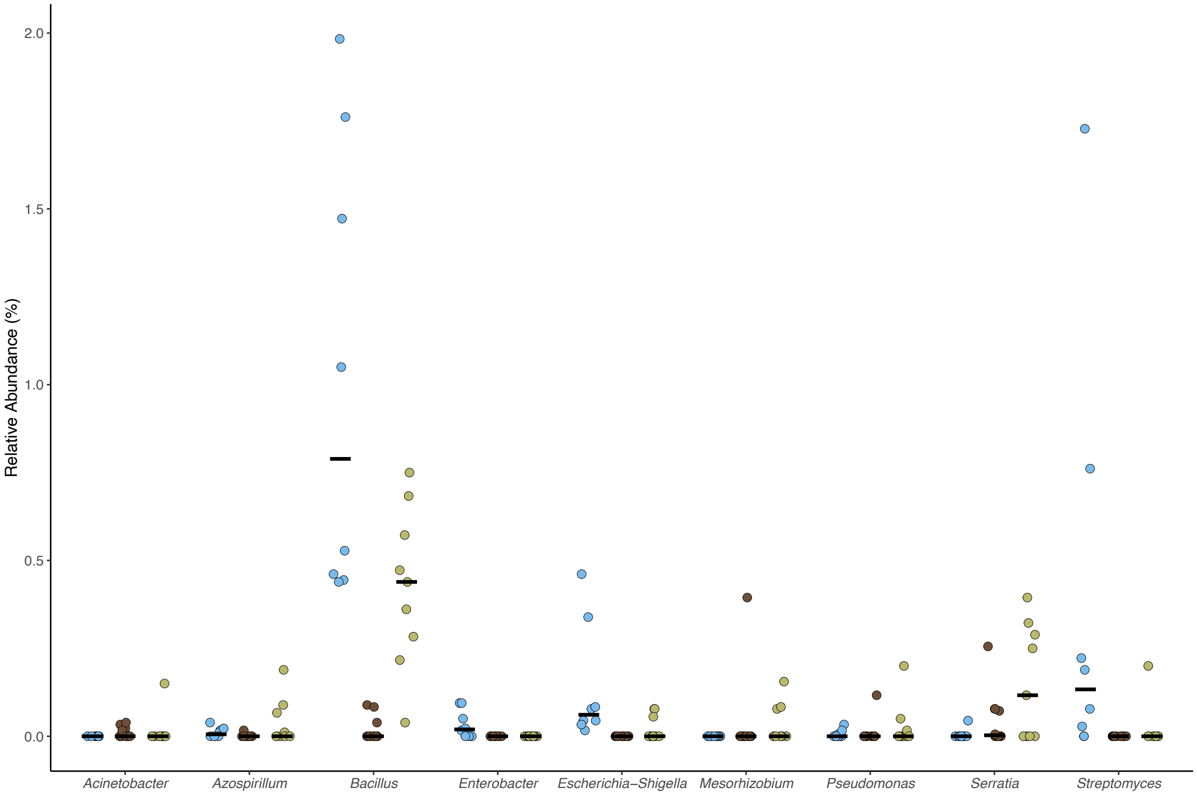

Supplementary Figure 8. Relative abundance of bacterial genera with capability of phosphate solubilization and iron acquisition in lithic communities. crossbars indicate the median relative abundance. Colored circles associated with microbial taxa indicate their presence in different lithic communities: endolithic communities within limestone (olive-green), epilithic communities on limestone (brown), and epilithic communities on sandstone (blue). Points refer to each sample.
